# Limited Benefit of Fish Consumption on Risk of Hip Fracture among Men in the Community-Based Hordaland Health Study

**DOI:** 10.3390/nu10070873

**Published:** 2018-07-06

**Authors:** Hanne Rosendahl-Riise, Gerhard Sulo, Therese Karlsson, Christian A. Drevon, Jutta Dierkes, Grethe S. Tell

**Affiliations:** 1 Department of Clinical Science, Faculty of Medicine, University of Bergen, 5020 Bergen, Norway; therese.e.karlsson@hotmail.com; 2 Center for Disease Burden, Norwegian Institute of Public Health, 5020 Bergen, Norway; Gerhard.Sulo@uib.no; 3 Department of Nutrition, Institute of Basic Medical Sciences, Faculty of Medicine, University of Oslo, 3017 Oslo, Norway; c.a.drevon@medisin.uio.no; 4 Center for Nutrition, Department of Clinical Medicine, Faculty of Medicine, University of Bergen, 5020 Bergen, Norway; Jutta.Dierkes@uib.no; 5 Laboratory Medicine and Pathology, Haukeland University Hospital, 5021 Bergen, Norway; 6 Department of Global Public Health and Primary Care, University of Bergen, 5021 Bergen, Norway; Grethe.Tell@uib.no

**Keywords:** diet, food frequency questionnaire, fish intake, hip fractures

## Abstract

Hip fractures have a high prevalence worldwide. Few studies have investigated whether fish consumption is associated with risk of hip fractures. The objective of the present study was to investigate the effect of fish intake on the subsequent risk of a hip fracture because of the low number of studies on this topic. A community-based prospective cohort study of 2865 men and women from Hordaland county in Norway, born between 1925–1927 and enrolled in the study in 1997–1999. Information on hip fracture cases was extracted from hospital records until 31 December 2009. Baseline information on the intake of fish was obtained from a semi-quantitative food frequency questionnaire. Cox proportional hazard regression models with death as a competing risk were used to evaluate the association of fish intake with risk of hip fracture. During a mean (SD) follow-up time of 9.6 (2.7) years, 226 hip fractures (72 in men, 154 in women) were observed. The mean (SD) fish intake was 48 (25) g/1000 kcal. The association between fish intake and risk of hip fracture was not linear and displayed a threshold, with low intake of fish being associated with an increased risk of hip fracture in men (HR (Hazard Ratio) = 1.84, 95% CI 1.10, 3.08). In this community-based prospective study of men and women, a low intake of fish was associated with the risk of a hip fracture in men.

## 1. Introduction

Hip fracture is a major public health challenge in aging populations worldwide with especially high prevalence in Caucasian populations [1]. Hip fractures substantially increase the risk of morbidity and death in older persons [2,3]. The one-year mortality is about 10–30 percent depending on sex, mental health, and comorbidity burden before the fracture [4,5,6,7]. Low bone mineral density (BMD) is the strongest single risk factor for hip fracture and it has been estimated that the risk ratio for hip fracture in men and women increases threefold for each standard deviation (SD) reduction in BMD [8,9]. However, as there are no good biomarkers or clinical signs of changes in BMD, a hip fracture is often the first sign of low BMD [9]. Postmenopausal women have a more drastic decrease in BMD than men of the same age do. Because women also have a longer life expectancy than men, the majority of low-energy hip fractures occur in women [9].

In addition to low BMD and sex, additional modifiable risk factors such as smoking [10], high alcohol consumption [11], low body weight [12,13], and low physical activity [14] are associated with an increased risk of hip fracture. Diet is discussed as another modifiable risk factor. Much focus in the past has been on single nutrients such as protein [15,16], calcium [17], vitamin D [18], vitamin K [19], and omega-3 polyunsaturated fatty acids (*n-*3 PUFAs) [20]. There is also some evidence that dairy products, as good sources of calcium [21], and a high intake of fruit and vegetables are associated with a lower risk of hip fracture [22,23]. Other food groups, including fish, have been investigated to a lesser degree. Because fish contains several potentially beneficial nutrients, including high quality protein, vitamin D, selenium, iodine, and *n-*3 PUFAs, it is an interesting food group with respect to prevention of hip fractures. Published articles provide mixed results on the association between fish intake and risk of hip fracture; two prospective studies reported a non-significant association [24,25], whereas a case-control study reported a protective effect of high fish intake [26]. In addition, studies have investigated fish as a part of a dietary pattern and reported beneficial but non-significant associations [22] or a beneficial significant effect [27,28]. Thus, the role on fish intake on hip fracture risk is uncertain.

Fish intake shows a high variation among populations, with Spain, Iceland, and Norway on top in the Western world. Total fish intake in these countries is about 50–70 g per day on average and is thus about 3–4 times that of central European countries [29] and the US [24,25].

Studying a Norwegian population of old men and women with among the highest hip fracture prevalence in the world, and with habitually high fish consumption, gives the unique opportunity to investigate total fish intake as well as sub-groups of fish. Thus, the main objective of this study was to investigate the relationship between total, lean, and fatty fish intake and the risk of hip fracture in the Hordaland Health Study (HUSK).

## 2. Materials and Methods

### 2.1. Subjects

We used data from the large community-based Hordaland Health Study (HUSK), Western Norway [30]. Participants were born between 1925–1927 and the baseline examinations were conducted during 1997–1999 [31]. Information on hip fractures was collected from the hospitals located in Hordaland County.

### 2.2. Dietary Assessment

The dietary assessment, questions related to dietary intake of fish and categorization of alcohol consumption, has previously been described in detail [32,33]. Briefly, habitual dietary intake was assessed using a 169-item food frequency questionnaire (FFQ) developed at the Department of Nutrition, Institute of Basic Medical Sciences, University of Oslo. The FFQ has been validated against weighted dietary records and against fatty acid composition in serum phospholipids [34,35,36]. Participants with an energy intake lower than 700 kcal or 800 kcal and higher than 3600 kcal or 4200 kcal for women and men, respectively, were removed from the analyses, leaving 2865 participants.

In addition to total fish intake (lean, fatty, and processed fish), fish intake was divided into fatty fish (herring, mackerel, salmon, trout, and fish used as spread) and lean fish (cod, pollock, and haddock). The multivariate nutrient density method was used for energy adjustments [37] of all dietary variables and presented as g/1000 kcal (foods and micronutrients) or energy percent (macronutrients). Total marine n-3 PUFA intake was calculated by adding eicosapentaenoic acid (EPA), docosapentaenoic acid (DPA), and docosahexaenoic acid (DHA).

One alcohol unit was defined in accordance with the Nordic Nutrition Recommendations 2012 [38]. Sex specific categories of alcohol intake was defined as: 0 = 0 g/day; 1 = women: >0–10 g/day; men: >0–20 g/day; 2 = women: >10–20 g/day; men: >20–30 g/day; 3 = women: >20 g/day; men: >30 g/day.

### 2.3. Hip Fractures

Collection of information on hip fractures and death has been previously described [39,40], along with a validation of the collection method [41]. Briefly, information on hip fractures was obtained from computerized records containing discharge diagnoses for hip fractures from all hospitalizations between the baseline examinations in HUSK through until 31 December 2009 at the six hospitals in Hordaland County, Norway. Participants were followed until they experienced their first hip fracture or died. A hip fracture was defined as the first fracture of the proximal femur during the observation period. Information on time of death was obtained from the Norwegian Population Register.

### 2.4. Covariate Assessment

Self-administered question provided information regarding current estrogen therapy, physical activity, and smoking. Physical activity was categorized as by Vinknes et al. [42]. The sum of the scores from categorization of light and hard physical activity was calculated. Categories for light physical activity were 0 (none), 0.25 (<1 h/week), 0.5 (1–2 h/week), or 1.0 (≥3 h/week) and for hard physical activity were 0 (none), 0.5 (<1 h/week), 1.0 (1–2 h/week), or 2.0 (≥3 h/week). Smoking habits were categorized as current smoker, former smoker, and never smoked. Measurements of plasma cotinine were used as a marker of recent nicotine exposure, and participants with cotinine levels ≥85 nmol/L were defined as smokers [43,44]. The psychical activity score and plasma cotinine were used in the multivariate models.

### 2.5. Ethics

HUSK was performed according to the declaration of Helsinki. All participants provided written informed consent. The Regional Committee for Medical and Health Research Ethics approved the study protocol (REC number: 2009/825).

### 2.6. Statistical Analyses

Continuous variables are presented as means and standard deviation and categorical variables as percentages. Differences in baseline characteristics and dietary intake across sex-specific quartiles were calculated by linear regression for continuous variables and logistic regression for dichotomous variables.

Fish intake (the exposure variable) was introduced in the analyses (a) in four categories (based on its sex-specific quartiles) and (b) as a dichotomous variable (comparing the lower quartile, Q1, to the upper three quartiles Q2 to Q4).

To visually explore the association between fish intake and risk of hip fracture, we used Cox proportional hazards regression model fitting fish intake using restricted cubic splines with three knots. Cox proportional hazards regression models with death as competing risk were used to estimate the risk of hip fractures across fish intake quartiles. The results are presented as hazard ratios (HRs) and 95% confidence interval (CI). Models were adjusted for sex, BMI, plasma concentration of cotinine ≥85 nmol/L, physical activity, and energy intake. Further adjustments for intake of vegetable, fruit, dairy, or meat, with vitamin D and calcium intake, or estrogen therapy (in women) were made, none of which changed the strength of the association. In addition to overall analyses, we also conducted sex-specific analyses. Statistical software SPSS for Windows version 25 (IBM, New York, NY, USA) and Stata Statistical Software (Release 15.0, College Station, TX, USA: Stata Corp LLC) were used and a two-sided *p*-value <0.05 was considered statistically significant.

## 3. Results

Out of 3327 eligible participants, information regarding food intake and hip fractures was available for 2865 (86%).

### 3.1. Baseline Characteristics and Dietary Intake

Mean (SD) follow-up time was 9.6 years (2.7). Total number of hip fractures was 226 (7.9%), 72 (5.5%) in men and 154 (9.9%) in women. During the study period, 448 men and 305 women died. Death as first event (without experiencing a hip fracture) was observed in 412 (31.3%) men and 257 (16.6%) women. Mean (SD) total fish intake as exposure variable of interest was 48 (25) g/1000 kcal in the entire cohort, with higher consumption in men (51 (25) g/1000 kcal) than in women (45 (25) g/1000 kcal). Lean fish was the largest fish category consumed, followed by fatty fish, and processed fish, both in men and women.

Characteristics of the total cohort stratified by sex-specific quartiles of total fish intake at baseline are presented in Table 1. Both men and women had increasing weight and BMI with higher fish intake. In women, the femoral neck BMD increased with increasing fish intake, but this was not evident in men. Self-reported smoking, plasma cotinine levels, and self-reported high alcohol consumption did not differ across quartiles of fish intake. 

Dietary intake of the cohort stratified by sex-specific quartiles of total fish intake at baseline is presented in Table 2. The energy adjusted protein intake (E %) was significantly higher in high consumers of fish compared to low consumers, both in men and women. The same was evident for total vitamin D intake, whereas calcium intake was lower in the high consumers of fish. Although few participants used fish oil supplements, there were more users of this supplement in high consumers of fish. A higher proportion of men with high fish consumption used cod liver oil as supplement compared to the low consumers. Of the food groups consumed, there was an increased intake of vegetables across quartiles in both men and women. In women, the high consumers of fish had a higher intake of fruit and berries compared to the low consumers. Compared to participants with low fish consumption, those with high consumption had lower intake of dairy products.

### 3.2. Fish Consumption and the Risk of Hip Fractures

The association of fish intake with hip fracture risk was first explored using fish intake as sex-specific quartiles with separate estimates for total, lean, and fatty fish. The analysis of total fish intake as sex-specific quartiles did not reveal any association between fish intake and the risk of hip fractures, neither in men nor in women (Table 3), nor in the total cohort (data not shown). This result did not change substantially after multiple adjustments. A competing risk analysis of mortality and hip fractures was performed; the results did not change materially (data not shown). A sensitivity analysis was also performed leaving out the non-consumers of lean and fatty fish, but this did not change the results. Separate analyses for lean and fatty fish showed no associations either (data not shown).

**Figure 1:**
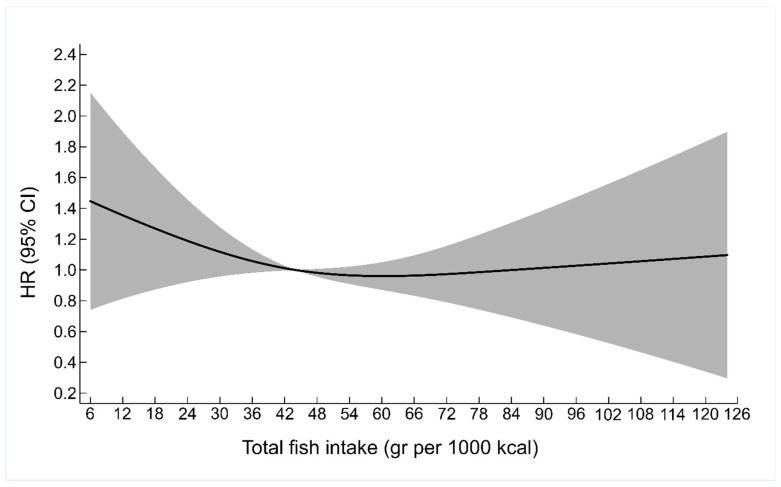
A restricted cubic spline exploring the non-linear association between total fish intake and the risk of hip fracture in the total cohort of men and women (70–74 years old at baseline) from the Hordaland Health Study. HR denotes Hazard Ratio, CI confidence interval.

The analysis was then extended to include a possible non-linear association of fish intake and hip fracture risk. Low fish intake was associated with higher hip fracture risk although not significantly (Figure 1). A higher risk of hip fractures was also observed when the lowest quartile of fish intake (Q1) was compared to higher fish intake (Q2–Q4) (Table 4). This association was, however, only significant in men. The results remained unchanged after multiple adjustments and competing risk analysis (data not shown).

## 4. Discussion

Our results suggest the existence of a threshold of fish intake below which the risk of hip fracture appears to increase in older men. This finding is not altered by multiple adjustments and examining death as a possible competing risk for hip fracture.

### 4.1. Comparison with Other Studies

Other prospective studies that have investigated the association with fish intake and the risk of hip fracture did not find significant associations [24,25,45]. A major difference among studies is the amount of fish consumed. In our study, the fish consumption was high. Participants in the previous studies would be categorized mainly in the lowest quartile of fish intake in our study [24,25,45]. However, comparison between studies is difficult because other scientists have categorized fish intake differently [24,25,26]. The amount of fish consumed in the present study was high enough to allow analysis of sub-groups of fish intake (lean and fatty fish) separately. However, no association with the risk of hip fracture was observed in the sub-groups. Other studies have performed sub-group analysis of fish intake, but the categorization differs. As an example, some studies included shellfish in the fish category [25,26]. Other differences between the studies include methodological differences in dietary assessment and adjustment for covariates, and assessment of servings. Another methodological issue is evaluation of fish intake as part of a general dietary pattern [22,27,28]. In addition to the studies mentioned above that have been conducted in predominantly Caucasian populations, there are also studies from Asia [26,46,47]. Asian and Caucasian populations differ genetically, but also in lifestyle and dietary intake. However, the main conclusion from the studies in Asian populations was that there is no or only a weak protective association between fish intake and risk of hip fracture.

Assessment of dietary data is challenging, and methodological differences may account for different findings. Fish intake in most of these studies was assessed using a FFQ [24,25,27,28]. However, in the European Prospective Investigation into Cancer (EPIC)-study FFQ, diet history, and 24 h recall [22] were used. In the Japanese cohort [46] a non-specified dietary questionnaire was used. Amounts of fish in these studies were defined either in servings/week [24,25], times/week or month [45], or above and below median intake [22,27], as part of a dietary pattern [47], portions/day [28], or not accounted for [46], which makes any comparison difficult. Moreover, only a few studies reported energy-adjusted fish intake, and those who did used the energy residual method [25,26,28,45,47].

### 4.2. Fish and Hip Fracture

When evaluating the risk associated with a single food group, one always has to take into account the interactions between food groups. High fish consumers had a higher intake of meat, vegetables, and in women, fruit and berries, and lower intake of dairy. In our study, high fish consumers had a higher intake of meat and vegetables, and among women, high fish consumers ate more fruit and berries and less dairy food. Similar results have been reported from a Spanish study that had a comparable fish intake to our study. Protective effects of high vegetable and fruit consumption have been reported on the risk of hip fracture [23,48]. For other food groups, there is some uncertainty regarding an association. This is especially true for dairy products, which is a good source of calcium, although the association with the risk of hip fracture is uncertain [49]. In the present study, adjustment for dairy intake did not change the results.

Fish is a good source of n3 PUFAs that have been proposed to be protective [20,25,50,51]. In our population of Western Norway, the association of fish and PUFA intake may have been modified by the high frequency of cod liver consumption. In addition to n3 PUFAs, cod liver oil is also a source of vitamin D. In Norway, it is recommended to those older than 65 years as a vitamin D supplement. In national surveys, about half of the population followed this advice [52]. However, many older persons do not consume cod liver oil throughout the year, but information on periods of supplement use is lacking in HUSK.

Similar to other studies [22,26], we observed a stronger association of fish intake with hip fractures in men than in women. The association of low intake with increased risk was only significant in men, which could be due to lower baseline risk in men and a larger variation in fish consumption. These results may suggest there are other factors aside from fish consumption that are important for the prevention of hip fracture. In the same population, we previously reported an association between fish intake and BMD in women [32] whilst investigating fish intake as a continuous variable and in sex-specific quartiles. However, this did not affect the association of fish intake and hip fractures. The same was evident for the Framingham Osteoporosis study reporting an association between fish intake and BMD [53] but not with the risk of hip fracture [25]. In the Cardiovascular Health Study [24], there was no association between fish intake and either BMD or hip fracture risk.

### 4.3. Strength and Limitations

The strength of our community-based study is its large sample size with a narrow age range (70–74 years), a high habitual fish consumption, and a long observation period. The fish intake reported in our study was similar to intake obtained in the Norwegian dietary survey from 1997 [52] of the 60–79 age group, suggesting that the estimate was valid. The main limitation was that fish intake and data on potential confounders were only assessed at baseline, and not during the follow-up. However, dietary habits seldom change rapidly, especially among older persons living at home [54]. Thus, the intakes reported are likely to represent habits extended throughout a longer period of life.

## 5. Conclusions

In this large community-based study of men and women with a high habitual fish consumption, we found a significantly increased risk of hip fracture among men in the lowest quartile compared to the upper three quartiles of fish intake.

## Figures and Tables

**Table 1:** Baseline (1997–1999) characteristics of men and women (age 71–74 years) by sex-specific quartiles of total fish intake. The Hordaland Health study.

	Women					*p* for Trend	Men					*p* for Trend
Total Fish, g/1000 kcal (min–max)	Total 1–263	1st Quartile (*n* = 387) 1–28	2nd Quartile (*n* = 387) 28–41	3rd Quartile (*n* = 388) 41–57	4th Quartile (*n* = 387) 57–263		Total 3–186	1st Quartile (*n* = 329) 3–33	2nd Quartile (*n* = 329) 33–48	3rd Quartile (*n* = 329) 48–65	4th Quartile (*n* = 329) 65–186	
Hip fracture incidence, (%)	9.9	11.6	10.1	9.8	8.3	0.128	5.5	7.9	3.6	5.2	5.2	0.234
Femoral neck BMD, g/cm^2^	0.763 (0.113)	0.746 (0.108)	0.769 (0.121)	0.766 (0.116)	0.772 (0.106)	0.016	0.901 (0.140)	0.895 (0.127)	0.892 (0.133)	0.913 (0.146)	0.907 (0.152)	0.157
BMI, kg/m^2^	26.2 (4.4)	25.8 (4.3)	26.1 (4.4)	26.6 (4.3)	26.3 (4.5)	0.028	26.0 (3.2)	25.8 (3.3)	25.7 (3.0)	25.9 (3.1)	26.5 (3.3)	0.008
Weight, kg	67.7 (11.8)	66.5 (11.6)	67.8 (11.5)	68.5 (11.6)	68.1 (12.4)	0.036	79.5 (11.1)	78.8 (11.2)	78.7 (10.7)	79.1 (10.7)	81.5 (11.6)	0.002
*Hard physical activity, %*												
≤1 h/week	79.7	81.6	78.1	77.0	82.3	0.900	63.9	67.4	62.8	62.9	62.3	0.209
>1 h/week	20.3	18.4	21.9	23.0	17.7	0.900	36.1	32.6	37.2	37.1	37.7	0.209
*Smoking habits, %*												
Current smoker	15.2	19.9	11.4	12.6	16.8	0.322	19.5	21.0	20.1	18.2	18.5	0.351
Former smoker	25.0	23.8	22.3	24.7	29.2	0.057	60.1	62.3	56.8	62.3	59.0	0.706
Never smoked	61.2	57.6	67.9	62.6	56.8	0.496	24.0	20.4	25.8	24.0	25.8	0.166
Cotinine ≥85 nmol/L, %	15.3	20.1	11.5	12.7	17.0	0.316	19.4	20.8	20.2	17.7	18.9	0.400
*Alcohol categories, %* ^1^												
None	55.5	64.1	55.3	53.1	49.4	<0.001	32.6	35.6	31.9	30.1	32.8	0.386
Low	39.8	32.6	39.0	43.0	44.7	<0.001	59.8	58.7	60.8	61.4	58.4	0.980
Moderate	4.1	2.8	5.2	3.6	4.9	0.305	3.7	2.4	3.6	3.6	5.2	0.079
High	0.6	0.5	0.5	0.3	1.0	0.455	3.9	3.3	3.6	4.9	3.6	0.655
Estrogen therapy (for women), %	14.8	13.4	14.0	16.8	15.2	0.309	NA	NA	NA	NA	NA	NA

Values represent means (SD). *p* for trend across quartiles of total fish intake; linear regression for continuous variables and logistic regression for dichotomous variables. ^1^ None: 0 g/day, Low: women: >0–10 g, men: >0–20 g, Moderate: women: >10–20 g, men: >20–30 g, High: women: >20 g, men: >30 g. BMD bone mineral density, BMI body mass index, NA not applicable.

**Table 2:** Baseline (1997–1999) dietary intake of men and women (age 71–74 years) by sex-specific quartiles of total fish intake. The Hordaland Health study.

	Women					*p* for Trend	Men					*p* for Trend
Total fish, g/1000 kcal (min–max)	Total 1–263	1st Quartile (*n* = 387) 1–28	2nd Quartile (*n* = 387) 28–41	3rd Quartile (*n* = 388) 41–57	4th Quartile (*n* = 387) 57–263		Total 3–186	1st Quartile (*n* = 329) 3–33	2nd Quartile (*n* = 329) 33–48	3rd Quartile (*n* = 329) 48–65	4th Quartile (*n* = 329) 65–186	
Total energy, kcal	1605 (491)	1557 (495)	1602 (475)	1646 (492)	1616 (499)	0.050	2059 (594)	2035 (599)	2135 (603)	2078 (553)	1988 (611)	0.180
Protein, E%	15.6 (2.9)	14.8 (2.1)	15.9 (2.0)	16.5 (2.1)	18.4 (2.4)	<0.001	15.5 (1.9)	14.5 (2.0)	15.6 (1.8)	16.3 (1.9)	18.0 (2.2)	<0.001
*n*-3 long chained PUFA, E%	0.1 (0.1)	0.1 (0.1)	0.1 (0.1)	0.1 (0.1)	0.2 (0.1)	<0.001	0.1 (0.1)	0. 1 (0.1)	0.1 (0.1)	0.1 (0.1)	0.2 (0.1)	<0.001
Vitamin D, g/1000 kcal ^1^	5.1 (4.3)	4.1 (4.2)	4.7 (4.0)	5.1 (4.1)	6.5 (4.4)	<0.001	5.6 (4.1)	3.9 (3.1)	5.3 (3.9)	6.1 (4.0)	7.2 (4.7)	<0.001
Calcium, g/1000 kcal ^1^	450 (138)	467 (151)	454 (132)	447 (134)	434 (131)	0.001	377 (124)	397 (144)	383 (124)	360 (107)	358 (116)	<0.001
*Supplements, %*												
Fish oil	7.0	3.9	6.7	5.9	11.4	<0.001	5.6	3.6	4.9	6.7	7.3	0.026
Cod liver oil	32.8	31.8	30.5	34.4	34.6	0.245	38.8	28.3	42.2	46.8	38.0	0.005
Vitamin D	2.6	2.8	2.3	2.6	2.6	0.885	1.8	0.9	3.0	0.9	2.4	0.462
Calcium	14.5	10.3	16.3	16.2	15.2	0.067	2.3	2.1	1.8	2.4	2.7	0.509
*Food intake, g/1000 kcal*												
Total fish	45 (25)	18 (6)	34 (4)	48 (5)	78 (22)	<0.001	51 (25)	23 (7)	41 (4)	55 (5)	85 (20)	<0.001
Lean fish	20 (17)	7 (5)	14 (7)	21 (9)	38 (22)	<0.001	23 (17)	9 (7)	17 (8)	25 (11)	41 (20)	<0.001
Fatty fish	13 (13)	5 (5)	9 (7)	13 (9)	23 (18)	<0.001	15 (13)	6 (6)	12 (8)	16 (10)	26 (17)	<0.001
Processed fish	12 (8)	6 (4)	11 (7)	14 (8)	17 (10)	<0.001	13 (9)	8 (6)	12 (6)	15 (8)	19 (11)	<0.001
Vegetables	115 (73)	94 (72)	114 (70)	117 (66)	135 (79)	<0.001	90 (60)	78 (59)	87 (57)	90 (52)	106 (69)	<0.001
Fruit and berries	151 (101)	138 (104)	158 (104)	152 (90)	157 (104)	0.032	114 (74)	109 (79)	118 (77)	116 (66)	113 (74)	0.542
Meat	40 (20)	34 (18)	41 (20)	42 (20)	42 (20)	<0.001	45 (21)	41 (21)	47 (20)	48 (20)	46 (21)	0.002
Dairy	193 (119)	217 (132)	201 (122)	187 (112)	166 (103)	<0.001	161 (106)	173 (118)	170 (106)	158 (97)	145 (102)	<0.001
Egg	10 (8)	9 (8)	10 (8)	9 (7)	10 (8)	0.520	9 (7)	8 (8)	10 (7)	9 (6)	9 (6)	0.742

Values represent means (SD). *p* for trend across quartiles of total fish intake; linear regression for continuous variables and logistic regression for dichotomous variables. ^1^ Intake of calcium and vitamin D includes use of supplements.

**Table 3:** Cox proportional hazards regression analysis of risk of hip fracture according to sex-specific quartiles (Q1–Q4) of baseline total fish intake among men (*n* = 1315) and women (*n* = 1548) in the Hordaland Health Study followed from inclusion in 1997–1999 (age 71–74 years) until 31 December 2009.

				Model 1			Model 2			Model 3		
		Total Fish Intake g/1000 kcal	*n* Hip Fracture/*n* Total	HR	95% CI	*p* for Trend	HR	95% CI	*p* for Trend	HR	95% CI	*p* for Trend
Men	Q1	23 (3–33)	26/329	1.56	(0.84, 2.87)	0.093	1.51	(0.82, 2.79)	0.091	1.75	(0.88, 3.47)	0.124
	Q2	41 (33–48)	12/329	0.68	(0.33, 1.43)		0.66	(0.31, 1.38)		0.79	(0.35, 1.78)	
	Q3	55 (48–65)	17/329	0.94	(0.48, 1.83)		0.91	(0.46, 1.79)		1.06	(0.50, 2.24)	
	Q4	85 (65–186)	17/329	1.00 (ref)			1.00 (ref)			1.00 (ref)		
Women	Q1	18 (1–28)	45/387	1.39	(0.87, 2.19	0.554	1.34	(0.85, 2.21)	0.649	1.47	(0.88, 2.44)	0.499
	Q2	34 (28–41)	39/387	1.23	(0.77, 1.96)		1.22	(0.76, 1.94)		1.37	(0.82, 2.30)	
	Q3	48 (41–57)	38/388	1.18	(0.74, 1.89)		1.20	(0.75, 1.92)		1.32	(0.76, 2.21)	
	Q4	78 (57–263)	32/387	1.00 (ref)			1.00 (ref)			1.00 (ref)		

Model 1: unadjusted; Model 2: adjusted for BMI (cont.); Model 3: adjusted for BMI (cont.), energy intake (cont.), nicotine exposure (plasma cotinine ≥ 85 nmol/L, yes/no), and physical activity score (none/low/moderate/high). HR denotes Hazard Ratio.

**Table 4:** Cox proportional hazards regression analysis of risk of hip fracture according to total fish intake comparing Q1 to Q2–Q4 for the total cohort (*n* = 2865), men (*n* = 1315), and women (*n* = 1548) from the Hordaland Health Study followed from inclusion in 1997–1999 (age 71–74 years) until 31 December 2009.

			Model 1			Model 2			Model 3		
		Total Fish Intake g/1000 kcal	HR	95% CI	*p*	HR	95% CI	*p*	HR	95% CI	*p*
Total cohort	Q1	21 (0.9–33)	1.40	(1.06, 1.85)	0.020	1.39	(1.05, 1.84)	0.022	1.36	(1.00, 1.85)	0.048
	Q2-Q4	57 (28–263)	1.00 (ref)			1.00 (ref)			1.00 (ref)		
Men	Q1	23 (3–33)	1.78	(1.10, 2.88)	0.018	1.77	(1.10, 2.87)	0.019	1.84	(1.10, 3.08)	0.021
	Q2-Q4	60 (33–186)	1.00 (ref)			1.00 (ref)			1.00 (ref)		
Women	Q1	18 (1–28)	1.23	(0.87, 1.74)	0.250	1.18	(0.83, 1.67)	0.350	1.20	(0.82, 1.75)	0.359
	Q2-Q4	53 (28–263)	1.00 (ref)			1.00 (ref)			1.00 (ref)		

Model 1: unadjusted; Model 2: adjusted for sex for the total cohort or BMI (cont.) for the different sex groups; Model 3: adjusted for BMI (cont.), physical activity score (none/low/moderate/high), nicotine exposure (plasma cotinine ≥ 85 nmol/L, yes/no), and energy intake (cont.) (and sex in the total cohort). HR denotes Hazard Ratio.
